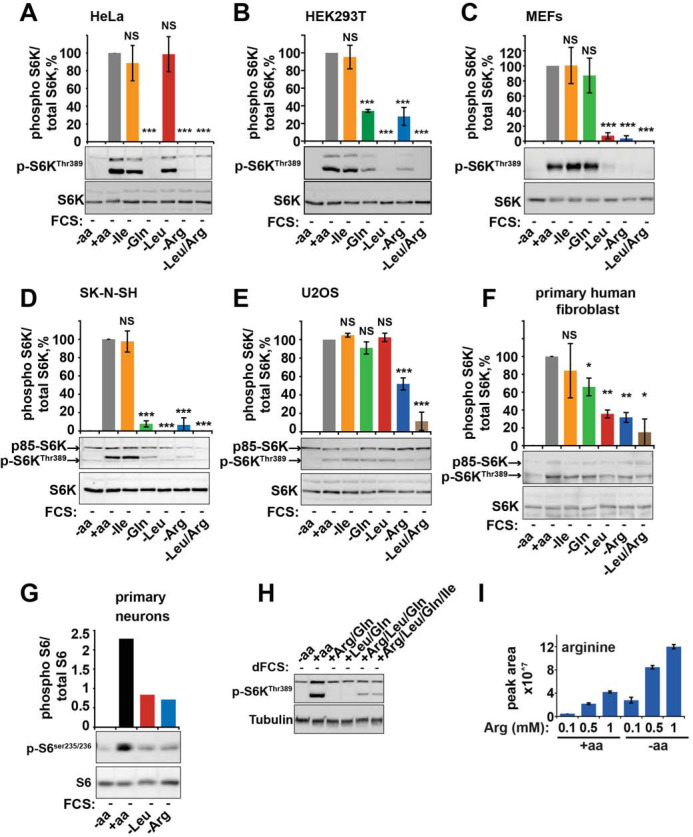# Correction: Control of TSC2-Rheb signaling axis by arginine regulates mTORC1 activity

**DOI:** 10.7554/eLife.65744

**Published:** 2020-12-16

**Authors:** Bernadette Carroll, Dorothea Maetzel, Oliver DK Maddocks, Gisela Otten, Matthew Ratcliff, Graham R Smith, Elaine A Dunlop, João F Passos, Owen Richard Davies, Rudolf Jaenisch, Andrew R Tee, Sovan Sarkar, Viktor I Korolchuk

Carroll B, Maetzel D, Maddocks ODK, Otten G, Ratcliff M, Smith GR, Dunlop EA, Passos JF, Davies OR, Jaenisch R, Tee AR, Sarkar S, Korolchuk VI. 2016. Control of TSC2-Rheb signaling axis by arginine regulates mTORC1 activity. *eLife* **5**:e11058. doi: 10.7554/eLife.11058.

Published 7, January 2016

It has come to our attention that in the published article, the loading control (total S6K) for Figure 1-figure supplement 1B was inadvertently duplicated from Figure 1-figure supplement 1D, a mistake that occurred during figure preparation. Figure 1-figure supplement 1B has now been corrected and no other changes were made to figures, figure legends or text.

The corrected Figure 1-figure supplement 1 is shown here:

**Figure 1:**
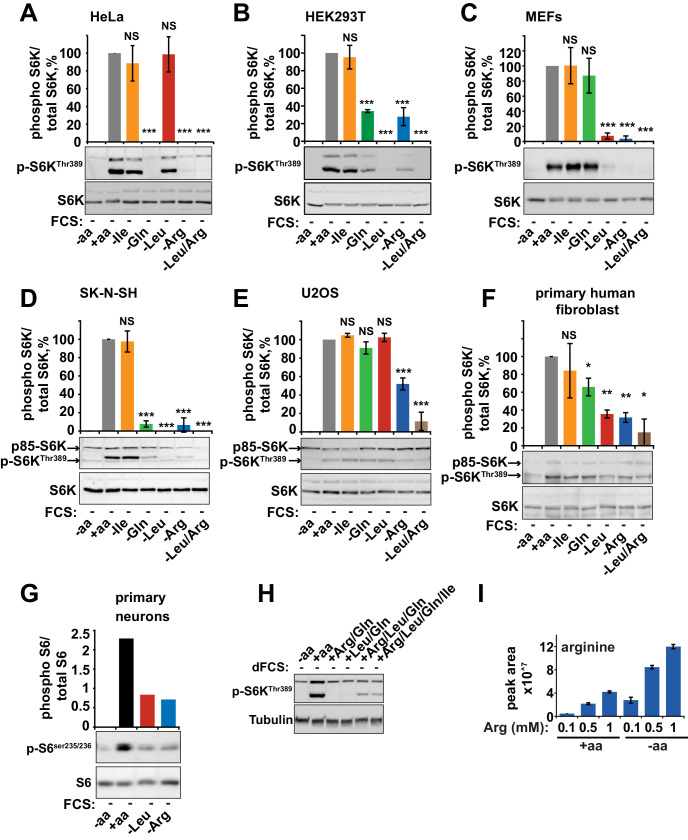


The originally published Figure 1-figure supplement 1 is also shown for reference:

**Figure 2:**