# Correction: Control of TSC2-Rheb signaling axis by arginine regulates mTORC1 activity

**DOI:** 10.7554/eLife.110979

**Published:** 2026-02-12

**Authors:** Bernadette Carroll, Dorothea Maetzel, Oliver DK Maddocks, Gisela Otten, Matthew Ratcliff, Graham R Smith, Elaine A Dunlop, João F Passos, Owen Richard Davies, Rudolf Jaenisch, Andrew R Tee, Sovan Sarkar, Viktor I Korolchuk

**Keywords:** Human

Carroll B, Maetzel D, Maddocks ODK, Otten G, Ratcliff M, Smith GR, Dunlop EA, Passos JF, Davies OR, Jaenisch R, Tee AR, Sarkar S, Korolchuk VI. 2016. Control of TSC2-Rheb signaling axis by arginine regulates mTORC1 activity. *eLife* **5**:e11058. doi: 10.7554/eLife.11058.

Published 7 January 2016

It has come to our attention via PubPeer that in the published article, there are two mistakes. The images from Figure 2C (MEFs -Arg+dFCS) were duplicated in Figure 3D (Rheb siRNA,+aa dFCS) and in Figure 3- supplement 2A the images from (Scr siRNA+aa dFCS) were duplicated in (Rheb siRNA,+aa+ dFCS). These mistakes occurred during figure preparation and do not affect the quantification associated with the figures. We apologise for these errors; both have now been corrected. No other changes were made to figures, figure legends or text.

The corrected Figure 3 (updated for panel D) is shown here:

**Figure 1:**
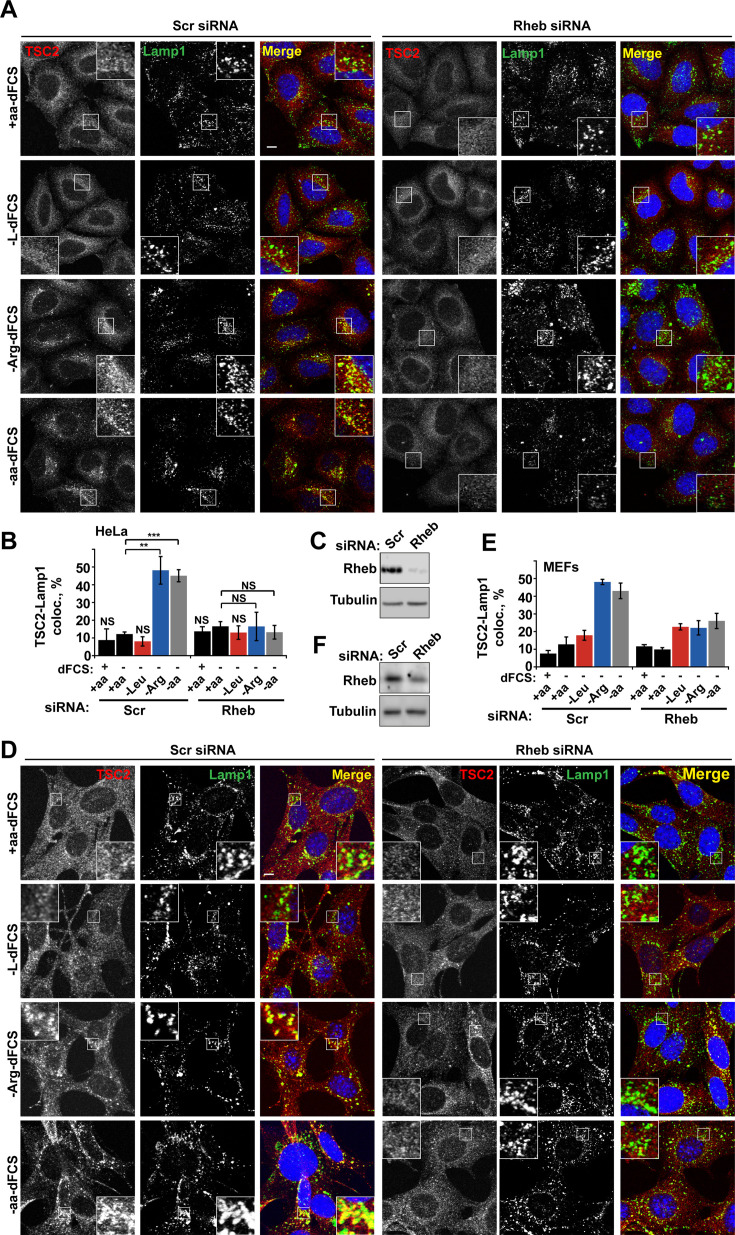


The originally published Figure 3 is shown for reference:

**Figure 2:**
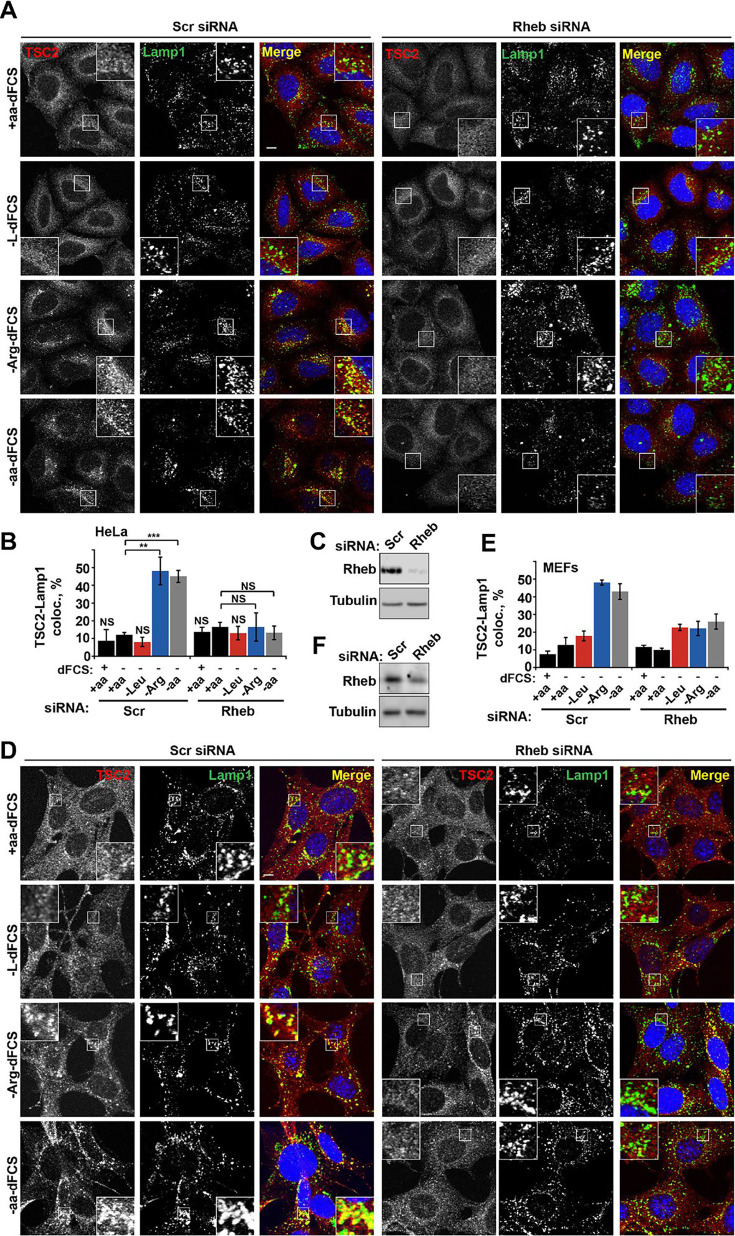


The corrected Figure 3—figure supplement 2 (updated for panel A) is shown here:

**Figure 3:**
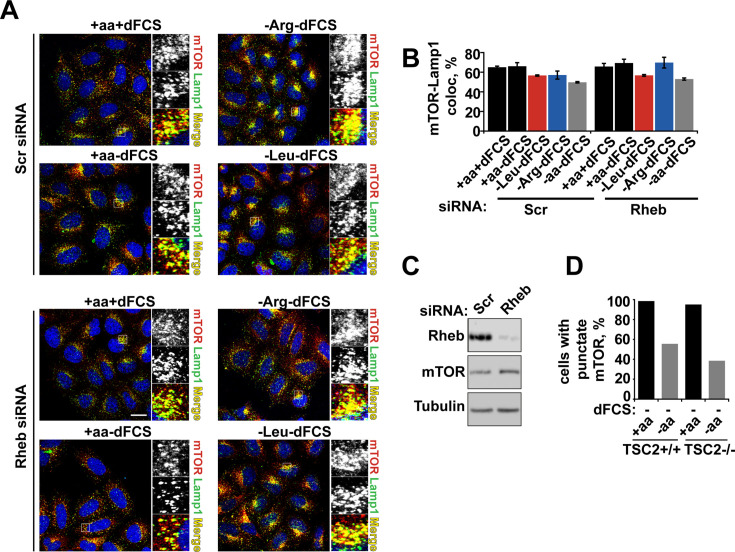


The originally published Figure 3—figure supplement 2 is shown for reference:

**Figure 4:**
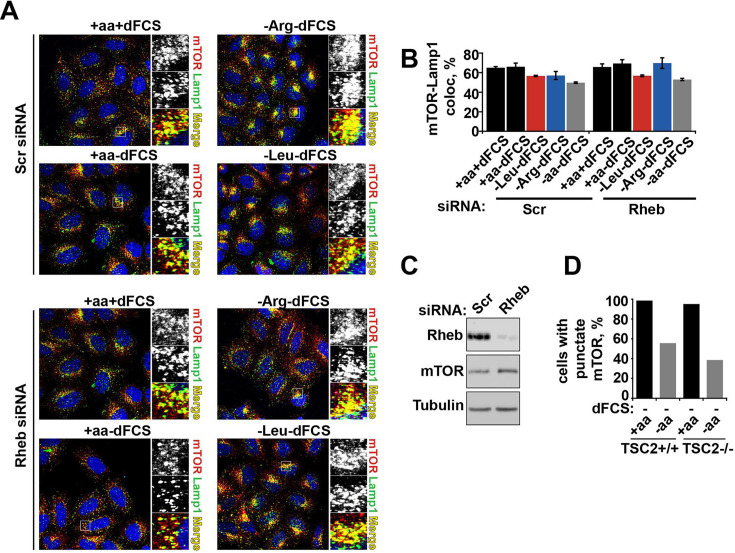


The article has been corrected accordingly.

